# Assisted Hatching Treatment of Piezo-Mediated Small Hole on Zona Pellucida in Morula Stage Embryos Improves Embryo Implantation and Litter Size in Mice

**DOI:** 10.3389/fcell.2021.746104

**Published:** 2021-10-20

**Authors:** Xin Hao, Yi-Tong Zhao, Kang Ding, Fang-Rui Xue, Xin-Yu Wang, Qi Yang, Zhe Han, Cheng-Guang Liang

**Affiliations:** State Key Laboratory of Reproductive Regulation and Breeding of Grassland Livestock, School of Life Sciences, Inner Mongolia University, Hohhot, China

**Keywords:** assisted hatching, embryo transfer, implantation, offspring, piezo

## Abstract

For *in vitro* produced embryos generated from *in vitro* fertilization (IVF) or intracytoplasmic sperm injection (ICSI) procedure, the intra- and extra-environmental factors during *in vitro* culture have significant impact on latter embryo development and fetus growth. Assisted hatching (AH), an effective approach to facilitate hatchability for *in vitro* generated embryos, is an essential step for successful embryo implantation in the uterus. However, regarding the different AH methods reported in clinical practice, it is still unknown whether zona pellucida (ZP) broken is based on AH applied in diverse stages of embryos affect implantation and fetal development. Here, piezo-mediated AH treatments were classified into four categories: (1) drilling one small hole (SH) with a diameter of 10 μm on ZP (SH); (2) drilling one large hole (LH) with a diameter of 40 μm on ZP (LH); (3) made a small area with diameter of 40-μm thinner on ZP [small area thinner (ST)]; (4) made a large area with a diameter of 80-μm thinner [large area thinner (LT)]. These four AH treatments were applied in different stage embryos including two-cell, four-cell, and morula. The most efficient AH approach was chosen according to the final hatch rate at 120 h after fertilization. We found that the approach of SH applied in morula-stage embryos obtained the highest hatch rate. To further investigate if this treatment has any side effect on later development after embryo transfer, we evaluated embryo implantation, gestational period, litter size, and growth. Our results showed that SH applied in morula-stage embryos could facilitate the implantation process and increase litter size. Meanwhile, this approach had no side effect on birth weight, growth, or gender ratio in the offspring. We conclude that drilling a SH on ZP in morula-stage embryos is an effective and reliable AH approach for *in vitro* cultured embryos in rodent. And this approach is worth further investigating in human-assisted reproductive technology.

## Introduction

For *in vitro* produced embryos, the procedures of gamete manipulation, such as *in vitro* fertilization (IVF) or intracytoplasmic sperm injection (ICSI), and the latter *in vitro* embryo culture conditions, such as medium components and blastocyst hatching, determine the final outcome of embryo implantation and fetus growth. Despite great achievements in IVF and ICSI in human-assisted reproduction, the implantation rate of transferred embryos remained low leading to lower pregnancy rate (around 33.7%) and even lower birth rate (around 27.1%) (Gunby et al., 2010). A previous study showed that exposure of embryos to the *in vitro* culture conditions impaired the ability of early embryo development and later blastocyst hatching (Gordon and Dapunt, 1993a). Hatching, which is essential for successful embryo implantation and establishment of early pregnancy, was achieved by a series of steps wherein the inner cell mass (ICM) is squeezed out of the zona pellucida (ZP) in the blastocyst stage (Hammadeh et al., 2011). To increase the hatchability of *in vitro* cultured embryos, assisted hatching (AH) was first proposed by Cohen in 1988 (Cohen et al., 1990).

Assisted hatching refers to any laboratory technique that artificially breaches or thins the ZP of an embryo prior to transfer into the uterus. The methods of AH include mechanical incision in the zona (Malter and Cohen, 1989; Sun et al., 2012), chemical zona drilling with acidic medium, chemical zona thinning (Yano et al., 2007), laser-assisted zona drilling or thinning (Hiraoka et al., 2009; Debrock et al., 2011), and piezo-assisted zona drilling or thinning (Nakayama et al., 1999; Ishibashi et al., 2013). Piezo-assisted zona drilling or thinning has been widely used for rodent oocyte microsurgical procedures due to its convenient and effective operation process. This approach carved a conical hole or thinned a limited area on ZP with vibratory movements produced by a piezo-electric pulse regulated by a controller (Nakayama et al., 1998).

Previous studies suggested that drilling a hole on ZP with diameter of 80 μm, rather than 6 or 20 μm, generated the best hatching result in the marmoset embryo (Ishibashi et al., 2013). Piezo-based zona thinning or drilling technique could facilitate blastocyst hatchability in humans (Nakayama et al., 1998), and this method is more effective in good quality embryos (Nakayama et al., 1999). Despite wide application of piezo-based AH, it is still not known if the embryo hatching rate and development is correlated with the detailed operating parameters, including the diameter of the drilling hole or thinning area on ZP, as well the embryo stages subjected to AH. Therefore, it is necessary to evaluate the effectiveness and safety according to implantation and fetal developmental potential regarding piezo-mediated AH.

In our study, to evaluate the effects of drilling or thinning size on ZP, mouse embryos that developed into t-cell, four-cell, or morula were used to assess the effectiveness and safety of AH. Our study will provide fundamental data for the employment of AH-based human-assisted reproductive technology in clinical practice.

## Materials and Methods

### Ethics Statement

Researches adhered to procedures consistent with the National Research Council Guide for the Care and Use of Laboratory Animals. All the experimental designs were approved by the Institutional Animal Care and Use Committee at Inner Mongolia University (Approval number: SYXK 2020-0006).

### Experimental Design

**FIGURE 1:**
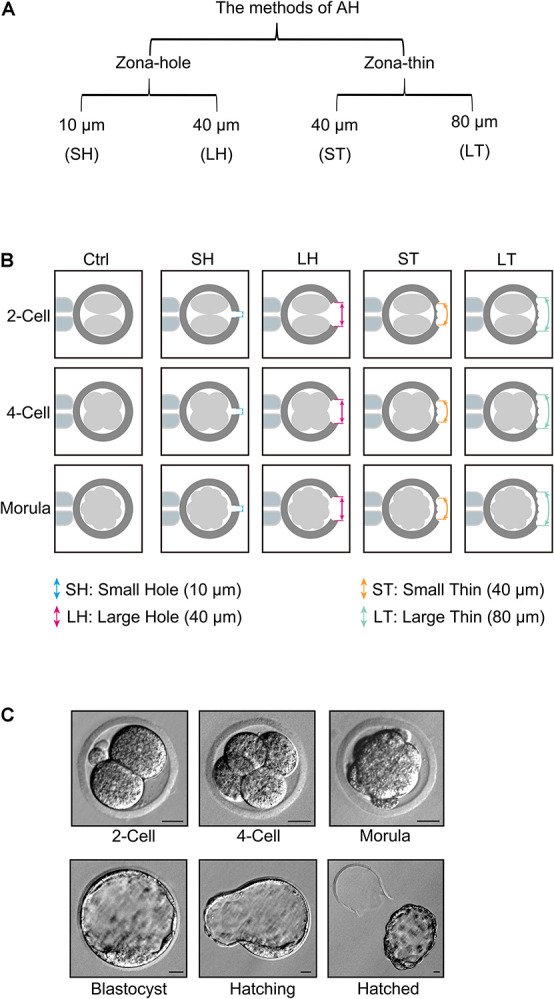
Piezo-mediated four kinds of assisted hatching (AH) treatments on different embryo developmental stages. **(A)** Different methods of AH by piezo, including zona-hole [small hole (SH): 10 μm; large hole (LH): 40 μm] and zona-thin [small area thinner (ST): 40 μm; large area thinner (LT): 80 μm] on zona pellucida (ZP). **(B)** Schematic of different AH treatments on different embryo developmental stages, including two-cell, four-cell, and morula. **(C)** Representative pictures of two-cell, four-cell, morula, blastocyst, hatching, or hatched embryos. Scale bar: 20 μm.

Piezo-mediated ZP broken were classified into four categories: (1) drilling one small hole (SH) with diameter of 10 μm (SH), (2) drilling one large hole (LH) with a diameter of 40 μm (LH), (3) made a small area with a diameter of 40 μm thinner [small area thinner (ST)], and (4) made a large area with a diameter of 80 μm thinner [large area thinner (LT; Figure 1A)]. All these four AH treatments were applied in different stage embryos including two-cell, four-cell, and morula. Embryos without any AH treatment were used as controls (Figure 1B). Representative pictures of two-cell, four-cell, morula, blastocyst, hatching, or hatched embryos are shown in Figure 1C.

### Animal Treatments

CD1 female mice at the age of 8 weeks and male mice at the age of 10 weeks were purchased from the Animal Research Centre at the Inner Mongolia University and housed in cages at 23°C with a photoperiod of 12-h light and 12-h darkness with free access to food and water.

Males with at least two mating records were used for vasectomy. Mice were anesthetized by intraperitoneal injection with ketamine solution (10 mg/ml) with a dosage of 0.01 ml/g and xylazine solution (2 mg/ml) with a dosage of 0.005 ml/g, followed by vasectomy surgery according to Bermejo-Alvarez et al. (2014). After recovery for 2 weeks, vasectomized mice were test-bred with 8-week-old females to ensure sterility prior to use. Vasectomy mice proved to be infertile were used for mating to prepare pseudopregnant recipients. After mating, plug-positive females were chosen for embryo transfer and recorded as 0.5-day postcoitus (dpc).

### Embryo Collection and Culture

Female mice were injected with 5 IU of pregnant mare serum gonadotropin (PMSG, SanSheng, Ningbo, China), followed by 5 IU of human chorionic gonadotropin (hCG, SanSheng, Ningbo, China) after 48 h. Superovulated females were mated with fertile males. *In vivo* developed two-cell stage embryos were flushed from oviduct with M2 medium containing 4% bovine serum albumin (BSA, Sigma-Aldrich) at 1.5 dpc. Embryos at the two-cell stage were allowed to develop to the four-cell or morula stage in Chatot–Ziomek–Bavister (CZB) medium supplemented with 3% BSA in a humidified atmosphere of 5% CO_2_ at 37°C. Embryos at the two-cell, four-cell, or morula stage were subjected to AH treatments and allowed to further develop to blastocyst for hatching analysis.

### Piezo-Mediated Assisted Hatching Treatments

For AH treatments, the holding pipette was prepared from a glass capillary tube with an outer diameter of 80–90 μm and an inner diameter of 10–12 μm. The piezo-driven operation pipette was prepared with a glass capillary tube to generate a blunt tip with 5 μm of the outer diameter. The parameter of piezo controller was set as speed = 3 and intensity = 3. For drilling a hole, ZP was incised by the micropipette through the continuous application of piezo pulses until the diameter of the hole achieved 10 or 40 μm. For thinning ZP, the operation pipette was subjected to continuous piezo pulse combined with movement along the ZP surface until the diameter of the thinning area achieved 40 or 80 μm. Operation on each embryo was finished within 30 s.

### Non-surgical Embryo Transfer

Embryo transfer mediated by the non-surgical embryo transfer (NSET) device was performed as described (Green et al., 2009; Bin Ali et al., 2014). Morula stage embryos with or without SH treatment were allowed to develop to blastocysts followed by embryo transfer. Briefly, each NSET device was loaded with 15 embryos treated with or without SH (labeled as W/SH or W/O SH, respectively). The tip was inserted to the edge of the speculum. The embryos were released into the uterus by pushing the plunger all the way down.

### Embryo Implantation Analysis

At 12.5 dpc, the recipient mice were anesthetized followed by tail vein injection with 1% Chicago Sky Blue dye. 3 min after injection, the numbers and size of implantation sites with obvious blue spots were observed and recorded after mice were sacrificed.

### Pregnancy and Offspring Assessment

At 16.5 dpc, the recipient mice with abdominal prominence were determined to be pregnant. Litter size and birth weight of pups were recorded immediately after delivery. The offspring were weaned at 3 weeks of age after gender distinction. The body weight of pups from 4 to 8 weeks of age was recorded.

### Statistical Analysis

Each experiment was performed at least in triplicate. At least 130 embryos were used for hatching analysis, and 120 embryos were transferred for each treatment. Two-way ANOVA with Tukey’s multiple *post hoc* comparison test in GraphPad Prism 7.0 (GraphPad Software Inc., San Diego, CA, United States) was used for statistically significant analysis in hatch rate, gestational period, body weight. One-way ANOVA with Tukey’s multiple *post hoc* comparison test in GraphPad Prism 7.0 was used for statistically significant analysis in pregnant rate, implantation sites per mouse, size of embryos, litter size, and survival pups. Chi-square test in the Microsoft Excel software (Microsoft Corporation, Washington, DC, United States) was used to analyze gender rate. Unpaired Student’s *t*-test in GraphPad Prism 7.0 was applied for the percent of implantation sites per transferred embryos, ratio of delivered pups. Results were shown as the mean ± SD. A value of ^∗^*p* < 0.05 was considered statistically significant.

## Results

### Embryo Development and Hatchability After Different Assisted Hatching Treatments on Same Stage Embryos

**FIGURE 2:**
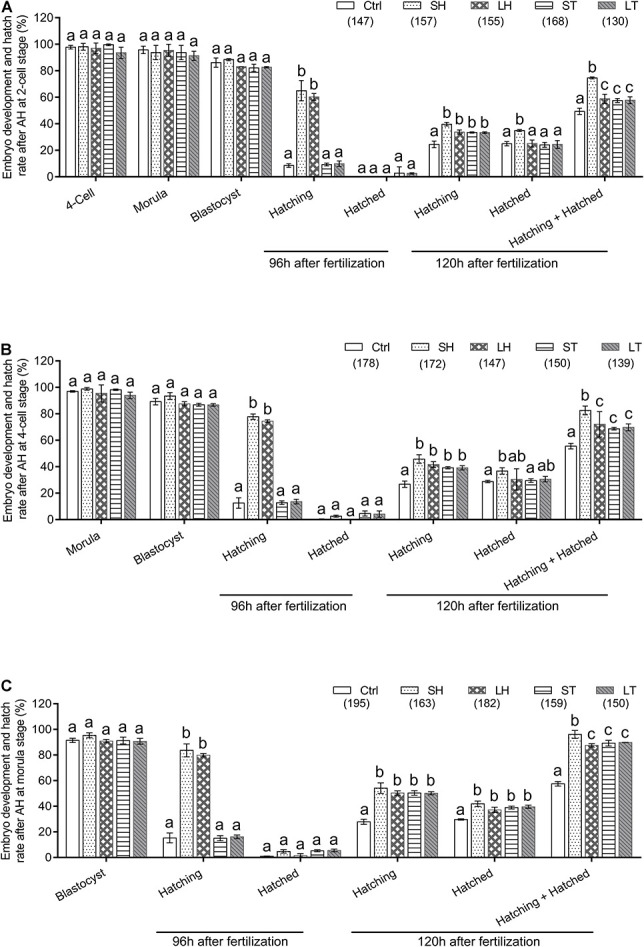
Embryo development and hatch rate after different AH treatments exerted on same stage embryos. **(A)** AH was performed on two-cell stage embryos. **(B)** AH was performed on four-cell stage embryos. **(C)** AH was performed on morula stage embryos. Embryos at two-cell stage were collected from oviduct after mating, followed by *in vitro* culture to allow to develop to four-cell and morula stages. The percent of hatching and hatched blastocyst at 96 and 120 h after fertilization were recorded. Data are presented as mean ± standard deviation (SD), and differences between groups were evaluated by two-way ANOVA with Tukey’s multiple *post hoc* comparison tests. Each experiment was performed at least in triplicate. The number of embryos assessed in each treatment is indicated in parentheses. Within the same stage embryos, percentages without a common letter were statistical different (*p* < 0.05).

When two-cell stage embryos were used, there was no statistically significant differences among the four AH treatments in terms of the percent of embryos developed at the four-cell, morula, and blastocyst (*p* > 0.05). Conversely, at the time of 96 h after fertilization, treatments of SH and LH have a much higher percent of hatching embryos than that of control, ST, and LT (*p* < 0.001). Very few embryos could complete the hatch process at 96 h after fertilization, even in the AH treatment groups (*p* > 0.05). However, AH treatments could significantly increase the percent of hatching embryos at 120 h after fertilization (*p* < 0.01). Significantly, treatment of SH could significantly increase the percent of hatched (*p* < 0.001) or hatching + hatched embryos (*p* < 0.001) (Figure 2A and Table 1).

**TABLE 1:** Embryo development and hatch rate after different assisted hatching (AH) treatments on 2-cell.

Treatment	4-cell(%)	Morula(%)	96 h after fertilization			120 h after fertilization		
			Blastocyst(%)	Hatching(%)	Hatched(%)	Hatching(%)	Hatched(%)	Hatching + hatched(%)
Ctrl	97.77 ± 1.35^a^	95.72 ± 2.79^a^	86.15 ± 3.52^a^	8.51 ± 1.32^a^	0.13 ± 0.22^a^	24.44 ± 2.53^a^	24.94 ± 1.56^a^	49.39 ± 2.32^a^
SH	98.14 ± 2.55^a^	93.79 ± 5.41^a^	88.44 ± 0.76^a^	64.98 ± 7.67^b^	0^a^	39.70 ± 1.37^b^	34.91 ± 0.71^b^	74.61 ± 0.74^b^
LH	96.94 ± 4.00^a^	95.38 ± 4.55^a^	83.04 ± 0.05^a^	60.22 ± 2.60^b^	0^a^	33.76 ± 1.59^b^	25.08 ± 2.64^a^	58.84 ± 3.41^c^
ST	99.63 ± 0.64^a^	93.79 ± 5.41^a^	82.17 ± 2.75^a^	9.30 ± 1.06^a^	2.77 ± 4.79^a^	33.45 ± 0.52^b^	24.10 ± 1.83^a^	57.55 ± 1.47^c^
LT	93.50 ± 4.21^a^	91.31 ± 3.42^a^	82.70 ± 0.50^a^	9.90 ± 2.02^a^	2.54 ± 0.51^a^	33.34 ± 0.59^b^	24.42 ± 2.93^a^	57.76 ± 2.57^c^

*Data are presented as mean ± standard deviation (SD) and were processed by Tukey’s multiple comparisons tests. Within the same embryo stage, percentages without a common letter were statistical different (*P* < 0.05).*

Similarly, when four-cell stage embryos were used, we did not observe any statistically significant differences for the percent of embryos developed at morula or blastocyst among the four AH treatments (*p* > 0.05). For the percent of hatching at 96 h after fertilization, treatments of SH and LH are much higher than that of control, ST, and LT (*p* < 0.001). In terms of hatched ratio at 96 h after fertilization, there was no statistical difference between the AH treatments and control (*p* > 0.05). When the embryos were checked at 120 h after fertilization, the AH treatments could significantly increase the percent of hatching embryos (*p* < 0.001), and the percent of hatched embryos is higher in the group of SH when compared with control (*p* < 0.05). When both hatching and hatched embryos were considered, treatment of SH could increase the percent when compared with other treatments (*p* < 0.001) (Figure 2B and Table 2).

**TABLE 2:** Embryo development and hatch rate after different AH treatments on 4-cell.

Treatment	Morula(%)	96 h after fertilization			120 h after fertilization		
		Blastocyst(%)	Hatching(%)	Hatched(%)	Hatching(%)	Hatched(%)	Hatching + hatched(%)
Ctrl	96.98 ± 0.60^a^	89.20 ± 2.47^a^	12.51 ± 3.93^a^	0.19 ± 0.34^a^	26.75 ± 2.43^a^	28.77 ± 0.84^a^	55.52 ± 2.12^a^
SH	98.86 ± 1.01^a^	93.44 ± 2.57^a^	77.81 ± 2.03^b^	2.51 ± 0.80^a^	45.79 ± 3.25^b^	36.74 ± 2.38^b^	82.53 ± 3.28^b^
LH	95.40 ± 6.47^a^	87.57 ± 1.59^a^	74.43 ± 1.04^b^	0^a^	41.62 ± 2.24^b^	30.39 ± 8.05^ab^	72.01 ± 9.60^c^
ST	98.17 ± 0.56^a^	86.87 ± 1.09^a^	12.79 ± 1.38^a^	4.63 ± 1.96^a^	39.17 ± 0.85^b^	29.52 ± 1.37^a^	68.69 ± 0.97^c^
LT	93.92 ± 2.43^a^	86.71 ± 0.97^a^	13.58 ± 1.74^a^	4.16 ± 2.44^a^	39.15 ± 1.64^b^	30.60 ± 2.06^ab^	69.75 ± 2.57^c^

*Data are presented as mean ± SD and were processed by Tukey’s multiple comparisons tests. Within the same embryo stage, percentages without a common letter were statistical different (*P* < 0.05).*

When morula stage embryos were used, all the embryos treated with or without AH have a similar ability to develop to blastocyst stage (*p* > 0.05). Embryos subjected to SH or LH have higher potential for hatching at 96 h after fertilization (*p* < 0.001), but very few of them could finish the hatch process (*p* > 0.05). AH treatments significantly increase the percent of hatching and hatched embryos at 120 h after fertilization (*p* < 0.001). Among the four AH treatments, SH obtained the highest percent of hatching + hatched embryos (*p* < 0.001) (Figure 2C and Table 3).

**TABLE 3:** Embryo development and hatch rate after different AH treatments on morula.

Treatment	96 h after fertilization			120 h after fertilization		
	Blastocyst(%)	Hatching(%)	Hatched(%)	Hatching(%)	Hatched(%)	Hatching + hatched(%)
Ctrl	91.40 ± 1.65^a^	15.29 ± 3.82^a^	0.99 ± 0.18^a^	27.83 ± 2.01^a^	29.69 ± 0.56^a^	57.52 ± 1.76^a^
SH	95.38 ± 1.94^a^	83.61 ± 5.03^b^	4.77 ± 1.36^a^	54.16 ± 4.11^b^	41.90 ± 2.16^b^	96.06 ± 3.12^b^
LH	90.88 ± 1.27^a^	79.86 ± 1.31^b^	1.44 ± 1.62^a^	50.34 ± 1.73^b^	37.18 ± 2.07^b^	87.52 ± 1.32^c^
ST	91.29 ± 2.66^a^	15.01 ± 1.99^a^	5.22 ± 0.78^a^	50.33 ± 1.78^b^	38.92 ± 1.12^b^	89.25 ± 2.16^c^
LT	90.69 ± 2.28^a^	16.10 ± 1.48^a^	5.49 ± 1.04^a^	50.14 ± 1.19^b^	39.58 ± 1.23^b^	89.72 ± 0.16^c^

*Data are presented as mean ± SD and were processed by Tukey’s multiple comparisons tests. Within the same embryo stage, percentages without a common letter were statistical different (*P* < 0.05).*

### Embryo Development and Hatchability After Same Assisted Hatching Treatment on Different Stage Embryos

**FIGURE 3:**
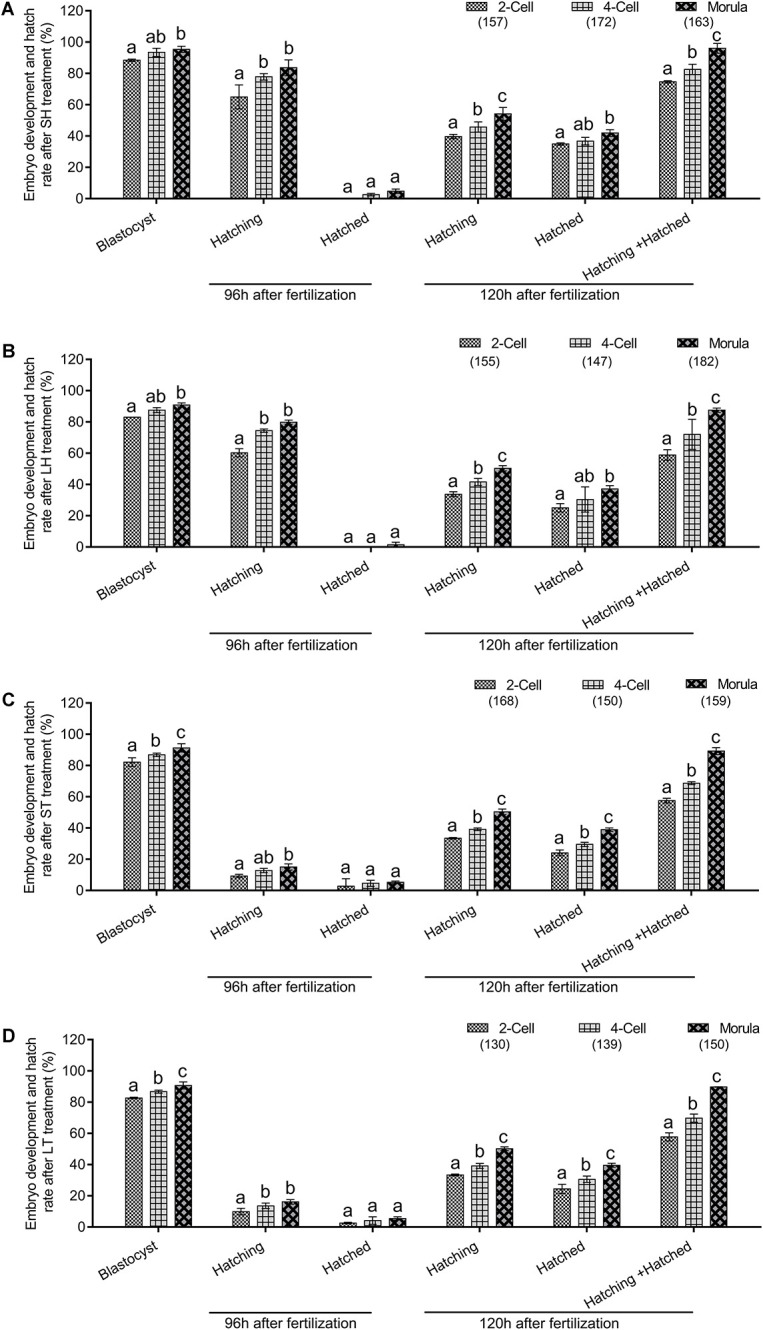
Embryo development and hatch rate after the same AH treatment exerted on different stage embryos. **(A)** SH treatment on different stage embryos. **(B)** LH treatment on different stage embryos. **(C)** ST treatment on different stage embryos. **(D)** LT treatment on different stage embryos. Embryos at two-cell stage were collected from oviduct after mating, followed by *in vitro* culture to allow to develop to four-cell and morula stages. The percent of hatching and hatched blastocyst at 96 and 120 h after fertilization were recorded. Data are presented as mean ± SD, and differences between groups were evaluated by two-way ANOVA with Tukey’s multiple *post hoc* comparison tests. Each experiment was performed at least in triplicate. The number of embryos assessed in each stage is indicated in parentheses. Within the same stage embryos, percentages without a common letter were statistically different (*p* < 0.05).

To further determine if AH has stage-dependent effects during early embryo development, the hatchability was re-analyzed to compare the effect of the same AH treatment on the different stage embryos. When SH treatment was applied, morula stage embryos have a higher percent of developing into blastocysts than those of two-cell (*P* < 0.05) and were comparable with that of four-cell (*p* > 0.05). Embryos of four-cell and morula treated with SH have a higher potential of hatching at 96 h after fertilization when compared with those of two-cell (*p* < 0.001). Embryos at each stage could hardly hatch out from zona at 96 h after fertilization, and even they were treated by SH. However, when the hatchability was checked at 120 h after fertilization, the morula stage embryos obtained the highest percent of hatching (*p* < 0.05), hatched (*p* < 0.05), or hatching + hatched (*p* < 0.01) when compared with those of two-cell (Figure 3A and Table 4).

**TABLE 4:** Embryo development and hatch rate after small hole (SH) treatment on embryos in different stages.

Embryos stage	96 h after fertilization			120 h after fertilizationh		
	Blastocyst(%)	Hatching(%)	Hatched(%)	Hatching(%)	Hatched(%)	Hatching + hatched(%)
2-cell	88.44 ± 0.76^a^	64.98 ± 7.67^a^	0^a^	39.70 ± 1.37^a^	34.91 ± 0.71^a^	74.61 ± 0.74^a^
4-cell	93.44 ± 2.57^ab^	77.81 ± 2.03^b^	2.51 ± 0.80^a^	45.79 ± 3.25^b^	36.74 ± 2.38^ab^	82.53 ± 3.28^b^
Morula	95.38 ± 1.94^b^	83.61 ± 5.03^b^	4.77 ± 1.36^a^	54.16 ± 4.11^c^	41.90 ± 2.16^b^	96.06 ± 3.12^c^

*Data are presented as mean ± SD and were processed by Tukey’s multiple comparisons tests. Within the same embryo stage, percentages without a common letter were statistical different (*P* < 0.05).*

Similar to the results of SH, when compared with two-cell, morula stage embryos treated with LH obtained better potential for blastocyst development (*p* < 0.05), hatching at 96 h (*p* < 0.001) or 120 h (*p* < 0.01) after fertilization, and the final hatched or hatching + hatched (*p* < 0.001) at 120 h after fertilization. When compared with four-cell, morula stage embryos treated with LH obtained better potential for hatching (*p* < 0.01) or hatching + hatched (*p* < 0.001) (Figure 3B and Table 5).

**TABLE 5:** Embryo development and hatch rate after; large hole (LH) treatment on embryos in different stages.

Embryos stage	96 h after fertilization			120 h after fertilization		
	Blastocyst(%)	Hatching(%)	Hatched(%)	Hatching(%)	Hatched(%)	Hatching + hatched(%)
2-cell	83.04 ± 0.05^a^	60.22 ± 2.60^a^	0^a^	33.76 ± 1.59^a^	25.08 ± 2.64^a^	58.84 ± 3.41^a^
4-cell	87.57 ± 1.59^ab^	74.43 ± 1.04^b^	0^a^	41.62 ± 2.24^b^	30.39 ± 8.05^ab^	72.01 ± 9.60^b^
Morula	90.88 ± 1.27^b^	79.86 ± 1.31^b^	1.44 ± 1.62^a^	50.34 ± 1.73^c^	37.18 ± 2.07^b^	87.52 ± 1.32^c^

*Data are presented as mean ± SD and were processed by Tukey’s multiple comparisons tests. Within the same embryo stage, percentages without a common letter were statistical different (*P* < 0.05).*

When ST treatment was applied in morula stage embryos, higher percent of blastocyst development and hatch at 120 h after fertilization (including hatching or hatched or both) were obtained when compared with two-cell or four-cell stage embryos (*p* < 0.05, Figure 3C and Table 6). The morula stage embryos also exhibited good potential in blastocyst development and hatchability at 120 h after fertilization when the LT method was applied (*p* < 0.05, Figure 3D and Table 7).

**TABLE 6:** Embryo development and hatch rate after small area thinner (ST) treatment on embryos in different stages.

Embryos stage	96 h after fertilization			120 h after fertilization		
	Blastocyst(%)	Hatching(%)	Hatched(%)	Hatching(%)	Hatched(%)	Hatching + hatched(%)
2-cell	82.17 ± 1.75^a^	9.30 ± 1.06^a^	2.77 ± 4.79^a^	33.45 ± 0.52^a^	24.10 ± 1.83^a^	57.55 ± 1.47^a^
4-cell	86.87 ± 1.09^b^	12.79 ± 1.38^ab^	4.63 ± 1.96^a^	39.17 ± 0.85^b^	29.52 ± 1.37^b^	68.69 ± 0.97^b^
Morula	91.29 ± 2.66^c^	15.01 ± 1.99^b^	5.22 ± 0.78^a^	50.33 ± 1.78^c^	38.92 ± 1.12^c^	89.25 ± 2.16^c^

*Data are presented as mean ± SD and were processed by Tukey’s multiple comparisons tests. Within the same embryo stage, percentages without a common letter were statistical different (*P* < 0.05).*

**TABLE 7:** Embryo development and hatch rate after large area thinner (LT) treatment on embryos in different stages.

Embryos stage	96 h after fertilization			120 h after fertilization		
	Blastocyst(%)	Hatching(%)	Hatched(%)	Hatching(%)	Hatched(%)	Hatching + hatched(%)
2-cell	82.70 ± 0.50^a^	9.90 ± 2.02^a^	2.54 ± 0.51^a^	33.34 ± 0.59^a^	24.42 ± 2.93^a^	57.76 ± 2.57^a^
4-cell	86.71 ± 0.97^b^	13.58 ± 1.74^b^	4.16 ± 2.44^a^	39.15 ± 1.64^b^	30.60 ± 2.06^b^	69.75 ± 2.57^b^
Morula	90.69 ± 2.28^c^	16.10 ± 1.48^b^	5.49 ± 1.04^a^	50.14 ± 1.19^c^	39.58 ± 1.23^c^	89.72 ± 0.16^c^

*Data are presented as mean ± SD and were processed by Tukey’s multiple comparisons tests. Within the same embryo stage, percentages without a common letter were statistical different (*P* < 0.05).*

In sum, for mouse preimplantation embryo AH, morula stage embryos treated with SH could obtain the best results of hatch.

### Small Hole Treatment in Morula Stage Embryos Improves Implantation and Pregnancy

Next, we want to know if increased hatchability induced by SH in morula stage embryos has any effects on embryo implantation and fetal development. Embryo implantation, gestational period, litter size, and growth were assessed and compared among the groups of natural conception (mating) and embryo transfer with or without AH treatment (abbreviated as W/SH and W/O SH, respectively).

**FIGURE 4:**
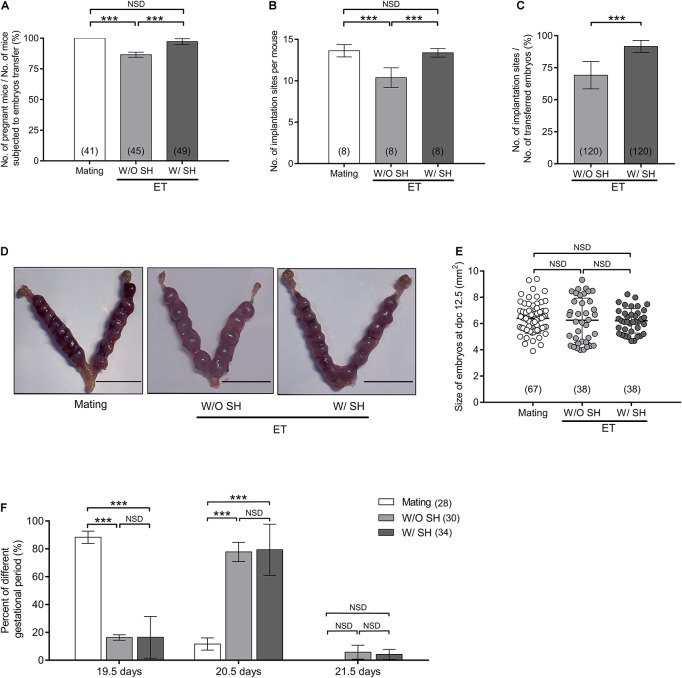
Small hole treatment in morula stage embryos improves embryo implantation and pregnancy. **(A)** Percent of pregnancy when mice subjected to embryo transfer were counted. **(B)** Number of embryo implantation sites per mouse. **(C)** Percent of implantation when the number of transferred embryos were counted. **(D)** Representative images of embryo implantation sites at day postcoitus (dpc) 12.5 in the group of mating, without SH or with SH. Scale bar: 1 cm. **(E)** Size of embryos at dpc 12.5. **(F)** Percent of different gestational periods. **(A,B,E)** Differences between groups were evaluated by one-way ANOVA with Tukey’s multiple *post hoc* comparison tests. **(C)** Differences between groups were evaluated by Student’s *t*-test. **(F)** Differences between groups were evaluated by two-way ANOVA with Tukey’s multiple *post hoc* comparison tests. Each experiment was performed at least in triplicate. The number of recipient mice **(A,B,F)** or embryos **(C,E)** assessed in each group is indicated in parentheses. Data are presented as mean ± SD; ***p < 0.001; NSD, no significant difference.

Pseudopregnant mice subjected to transfer of embryos W/O SH treatment have impaired pregnant outcome and implantation sites when compared with those of the mating group (*p* < 0.001), illustrating a long-term *in vitro* culture, or the embryo transfer operation affects embryo implantation. Conversely, AH treatment in the morula stage embryos could reverse the side effect of long-term *in vitro* culture, and increase the rate of pregnancy and number of implantation sites, which are comparable with that of the mating group (*p* > 0.05) (Figures 4A–C). Typical images of embryo implantation sites are shown in Figure 4D. However, AH treatment does not affect the size of embryos at dpc 12.5 (Figure 4E). Due to the delay of embryo development *in vitro*, the gestational period is increased when compared with the mating group. Most pseudopregnant mice subjected to embryo transfer need 20.5 days before laboring. AH treatment in *in vitro* cultured embryos could not shorten the gestational period (Figure 4F).

### Small Hole Treatment in Morula Stage Embryos Increases Litter Size Without Affection on Gender Ratio and Offspring Growth

**FIGURE 5:**
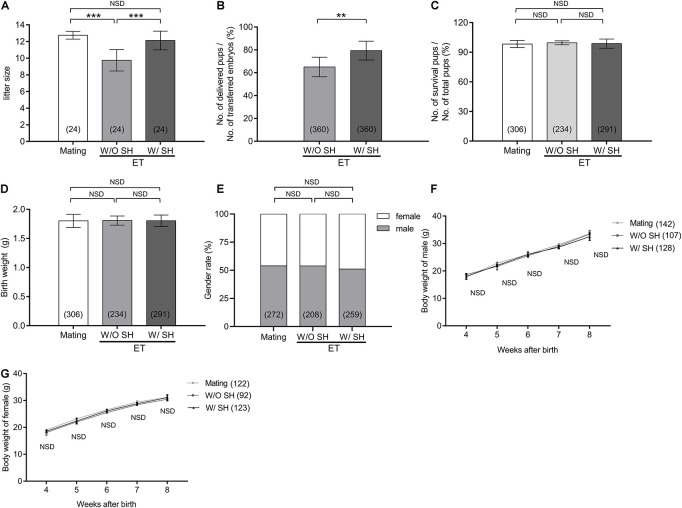
Small hole treatment in morula stage embryos increases litter size without affection on gender ratio or offspring growth. **(A)** Litter size. **(B)** The ratio of delivered pups to transferred embryos. **(C)** The ratio of survival pups to total delivered pups. **(D)** Birth weight. **(E)** Gender rate. **(F)** Body weight of male offspring from age of 4 to 8 weeks. **(G)** Body weight of female offspring from age of 4 to 8 weeks. **(A,C,D)** Differences between groups were evaluated by one-way ANOVA with Tukey’s multiple *post hoc* comparison tests. **(B)** Differences between groups were evaluated by Student’s *t*-test. **(E)** Differences between groups were evaluated using chi-square test. **(F,G)** Differences between groups were evaluated by two-way ANOVA with Tukey’s multiple *post hoc* comparison tests. Each experiment was performed at least in triplicate. The number of recipient mice **(A)**, transferred embryos **(B)**, or offspring **(C–G)** assessed in each group is indicated in parentheses. Data are presented as mean ± SD; **p < 0.01; ***p < 0.001; NSD, no significant difference.

The average litter size from the mating group is 12.75, which is significantly higher than that in the W/O SH group (9.75, *p* < 0.001). Increased litter size in the group of W/SH (12.13) is comparable with that of the mating group and much higher than that in the W/O SH group (*p* < 0.001), demonstrating that the AH approach could facilitate embryo implantation and delivery (Figure 5A). Since the litter size is also determined by the number of transferred embryos, we compared the percent of delivered pups in which the number of transferred embryos were used as denominator. As expected, when 15 embryos were transferred to each surrogate mother, SH treatment could significantly increase the percent of delivery (Figure 5B). To evaluate if AH has any side effects on the health of offspring, percent of survival pups, birth weight, and gender rate were compared among the three groups. Our results showed that AH had no effects on offspring survival, birth weight, or gender rate (Figures 5C–E). Furthermore, long-term effect of AH on the offspring was evaluated by detecting the body weight increase in males and females from 4 to 8 weeks after delivery. Our results showed that AH or embryo transfer itself does not affect the body weight increase, proving the safety of SH treatment on morula stage embryos in mice (Figures 5F,G).

## Discussion

In this study, by comparing different assisted hatch protocols in various developmental stages of preimplantation embryos, we conclude that morula stage embryos subjected to piezo-mediated SH treatment could obtain the best results in terms of hatchability, embryo implantation, and offspring development after transfer by non-surgical approach in mice. This approach has no side effects on gender determination or offspring growth.

Assisted reproductive technology has been developing to advance during recent decades. A variety of techniques have been used to assist embryo hatching, including mechanical dissection (Cohen et al., 1990) or chemical digestion (Cohen et al., 1992) on ZP. Besides, laser-AH (Alteri et al., 2018) and piezo-assisted treatments (Nakayama et al., 1998) were also reported to promote the hatching process. Although mechanical incision in ZP is a simple procedure, it may have profound consequences for embryonic development. For instance, microorganisms, viruses, and cytotoxins present in the insemination suspension could invade and infect the embryos *via* the artificially produced gap in ZP. In addition, blastomeres may be lost through LHs, causing embryonic death or vesiculation (Gordon et al., 1988). Moreover, immune cell invasion through gaps in the ZP may cause embryonic death, prior to the formation of tight junctions (Willadsen, 1979). In terms of chemical digestion in which acidified Tyrode’s solution was commonly used, both partial thinning and circumferential thinning on ZP were reported (Khalifa et al., 1992; Gordon and Dapunt, 1993b). Chemical digestion on ZP has been performed widely because of technical ease and relative low cost. However, this technique may have risks for adverse effects. Embryos could be damaged by acidic or enzymatic solutions. Blastomeres could separate from embryos during cleavage, and bacterial infection within the uterine cavity could occur (Nichols and Gardner, 1989). As an alternative method, laser-mediated ZP opening represents an ideal tool for microsurgical procedures, as the energy is easily focused on the targeted area, producing an ideal and precise hole (Alteri et al., 2018), but this protocol may generate potential harm to embryos by heat shock. Also, UV radiation is a potential factor that induce the mutagenic. Thus, the laser instrument designed for clinical practice must consider the potential thermal effects and should minimize pulse duration and laser intensity (Douglas-Hamilton and Conia, 2001).

Piezo-micromanipulated AH treatments have been used for microsurgical procedures due to its convenient and effective operation process. This approach carved a conical hole or thinned a limited area on ZP with vibratory movements produced by a piezo-electric pulse regulated by a controller (Nakayama et al., 1998). The ZP of embryos was easily carved by a piezo-micromanipulator without any additional treatments, such as hypertonic solution use or subsequent successive washing procedures used in the partial ZP dissection method (Nakagata et al., 1997). Previous studies suggested that the rates of hatching and hatched blastocyst were significantly higher in ZP thinning combined with the drilling group than in the control group, which demonstrate that the technique by piezo is useful for AH (Nakayama et al., 1998). The clinical rates of pregnancy and implantation were also improved by using piezo-micromanipulator-mediated AH (Nakayama et al., 1999). In our study, the best hatchability was obtained by piezo-mediated SH treatment, which was likely due to the small gap on ZP that caused little damage to the integrity of embryos. Another possible factor for the higher hatchability by piezo-mediated SH treatment may be that blastocyst was easily squeezed from the SH on ZP. Although this approach has been verified for its reliability in rodents, the efficiency of piezo-mediated SH method should be verified in primates and humans.

Besides, it was still a question in which stage embryos could the treatment by piezo generated the best hatch results. Zygotic gene activation (ZGA) occurs at the two-cell stage embryos in which a class of gene expression transiently increases (Davis et al., 1996). AH manipulating at this stage may cause adverse effects on gene expression and later embryo development. Previous findings showed that each cell in the four-cell stage embryos differ in their developmental fate and potency (Broquet et al., 1975; Piotrowska et al., 2001; Fujimori et al., 2003; Piotrowska-Nitsche et al., 2005). Minor damage induced by piezo in each cell would amplify its consequences in the latter development. Meanwhile, large-scale epigenetic modification in this developmental stage is more susceptible to mechanical manipulation (Torres-Padilla et al., 2007). Due to the volume expansion in the blastocysts, the ICM or trophoblast cells bonded tightly with ZP. Therefore, any AH process was likely to cause inner structure damage (Milki et al., 2000). Although the eight-cell stage embryos were commonly used for AH treatment in the clinic, it will be easier and safer to perform AH on the morula, a stage with better tolerance to mechanical manipulation. Morula stage embryos established extensive cell junction to form a more compact structure when compared with eight-cell stage embryos. Extensive cell junction in morula embryos excludes the possibility of losing an individual blastomere (Tao et al., 2001). Furthermore, the compact structure in the morula stage generates larger perivitelline space, which may be invulnerable to mechanical damage during AH manipulation (Li et al., 2013). Thus, morula, but not eight-cell stage, embryos were chosen for AH treatment in our study. Our study confirmed that conducting AH treatment in morula stage embryos would obtain the best outcome. This may due to a relative larger number of cells in morula stage embryos far from ZP could endure the mechanical damage on the individual cells, which could be compensated and replaced by other cells.

Embryo transfer was conducted to verify its influence on embryo implantation and offspring growth. Appropriate communication between the developing conceptus and maternal endometrium is essential for the establishment and maintenance of pregnancy (Sanchez et al., 2018). The endometrium is receptive to the embryos for a specific period of time known as the window of implantation (WOI). During this period, the endometrium shows a specific gene expression profile suitable for endometrial function evaluation (Enciso et al., 2021). Assisted hatching may enhance embryo implantation not only by mechanically facilitating the hatching process but also by allowing earlier embryo–endometrium contact. Such early contact may enhance embryonic development potential and may optimize synchronization between embryos and endometrium, resulting in improved implantation efficiency (Liu et al., 1993). Our results did not show any affection on gender ratio after AH treatment, neither on the birth weight or latter growth, illustrating that piezo-mediated SH is safe. However, the gestational period was delayed when compared with natural mating. This is probably due to the delayed embryo development *in vitro*, but not by the AH treatment, since embryos cultured *in vitro* without any AH treatment exhibited a similar extended gestational period (Uppangala et al., 2016).

In a previous study, the technique of partial ZP digestion was applied when frozen mouse spermatozoa were used for IVF. Although the fertilization rate increased upon ZP digestion, the birth rates were not improved (Fan et al., 2009). In humans, the pregnancy outcomes were not improved even AH was applied before vitrified-warmed blastocyst transfer (Ng et al., 2020), and zona drilling does not improve IVF results in cases of severe semen alterations. These findings contradict our results that AH facilitates offspring birth.

However, these controversial results can be explained by the various endogenous and exogenous factors that may determine the final birth. First, the sperm or oocyte or embryo experienced frozen–thaw treatment, which may disable the effect of AH treatment. A Systematic study in bovine demonstrated that IVF-derived embryo formation was impaired due to ZP changes after oocyte cryopreservation (Mavrides and Morroll, 2005). Damage of oocyte ZP or sperm membrane induced by cold stress is irreversible, and the final birth rate cannot be improved by simple AH treatment. Second, in most studies, AH was performed in very early developmental stages (oocyte, two-cell, or eight-cell) or in blastocyst. The narrow perivitelline space in these stages increase the potential for laser or piezo-induced cytotoxic damage during ZP broken. In our study, morula stage embryos were chosen for SH treatment. Morula compaction generates a larger perivitelline space. We proposed that the enlarged perivitelline space will minimize laser-induced thermal or piezo-induced mechanical damage in cells. Our hypothesis was proven by a previous study conducted by Li et al. (2013). They found that laser-zona drilling-assisted IVF has a significantly lower birth rate when compared with that of embryos derived by regular IVF. On the contrary, the birth rate could be significantly increased by enlarging the perivitelline space upon the addition of 0.25 M sucrose to the culture medium.

The final birth rate was determined by various intra- and extra-environmental factors including hatching method, extent of AH, embryo transfer status, conception mode, and previous failure history. Incomparable conditions or operation procedures in different labs will influence the final effect of AH treatment. Nevertheless, a systematic review and meta-analysis elucidated that AH remarkably improved clinical pregnancy as well as multiple pregnancy rates in comparison with the control. Moreover, the rates of clinical pregnancy and multiple pregnancy significantly increased among women who received ICSI or AH that the zona was completely removed. In sum, this meta-analysis proved that AH was closely related to an improved possibility of achieving clinical pregnancy or multiple pregnancy (Li et al., 2016).

Piezo-mediated SH treatment on morula stage embryos obtained the best hatchability and improved implantation, pregnancy, and litter size while without any affection on gender ratio and offspring growth. Thus, AH treatment based on piezo could be an effective and reliable approach in rodents. However, in primates, especially in humans, this AH approach still needs further investigation. We did not examine if drilling at the blastocyst stage embryos could obtain better results than other stages of development, nor did we compare the methods of laser-assisted approach with that of the piezo approach. Thus, it is worth to set up an investigation in order to find the best approach for the personalized application of AH in human-assisted reproduction.

## Data Availability Statement

The original contributions presented in the study are included in the article/Supplementary Material, further inquiries can be directed to the corresponding author.

## Ethics Statement

The animal study was reviewed and approved by Researches adhered to procedures consistent with the National Research Council Guide for the Care and Use of Laboratory Animals. All the experimental designs were approved by the Institutional Animal Care and Use Committee at Inner Mongolia University (Approval number: SYXK 2020-0006).

## Author Contributions

C-GL, XH, and Y-TZ conceived and designed the experiments and analyzed the data. XH, Y-TZ, KD, F-RX, X-YW, QY, and ZH performed the experiments. XH, Y-TZ, and C-GL wrote the manuscript. All authors contributed to the article and approved the submitted version.

## Conflict of Interest

The authors declare that the research was conducted in the absence of any commercial or financial relationships that could be construed as a potential conflict of interest.

## Publisher’s Note

All claims expressed in this article are solely those of the authors and do not necessarily represent those of their affiliated organizations, or those of the publisher, the editors and the reviewers. Any product that may be evaluated in this article, or claim that may be made by its manufacturer, is not guaranteed or endorsed by the publisher.

## Supplementary Material

Supplementary Video 1 Representative small hole (SH) treatment was performed in 2-cell stage embryo. SH treatment in 4-cell or morula stage embryos is similar with that in 2-cell stage embryos. Scale bar: 50 μm.

Supplementary Video 2 Representative large hole (LH) treatment was performed in 2-cell stage embryo. LH treatment in 4-cell or morula stage embryos is similar with that in 2-cell stage embryos. Scale bar: 50 μm.

Supplementary Video 3 Representative small area thinner (ST) treatment was performed in 2-cell stage embryo. ST treatment in 4-cell or morula stage embryos is similar with that in 2-cell stage embryos. Scale bar: 50 μm.

Supplementary Video 4 Representative large area thinner (LT) treatment was performed in 2-cell stage embryo. LT treatment in 4-cell or morula stage embryos is similar with that in 2-cell stage embryos. Scale bar: 50 μm.
